# Surgery for Cardiac Arrhythmias: Past, Present, Future

**DOI:** 10.5041/RMMJ.10516

**Published:** 2024-01-19

**Authors:** Stephen D. Waterford, Niv Ad

**Affiliations:** 1 University of Pittsburgh Medical Center, Department of Cardiothoracic Surgery, Pittsburgh, Pennsylvania, USA; 2 Johns Hopkins University, Department of Surgery, Division of Cardiac Surgery, Baltimore, Maryland, USA

**Keywords:** Atrial fibrillation, cardiac arrhythmias, cardiac surgery, inappropriate sinus tachycardia

## Abstract

There is a rich history of surgery for cardiac arrhythmias, spanning from atrial fibrillation and Wolff–Parkinson–White syndrome to inappropriate sinus tachycardia and ventricular tachycardia. This review describes the history of these operations, their evolution over time, and the current state of practice. We devote considerable time to the discussion of atrial fibrillation, the most common cardiac arrhythmia addressed by surgeons. We discuss ablation of atrial fibrillation as a stand-alone operation and as a concomitant operation performed at the time of cardiac surgery. We also discuss the emergence of newer procedures to address atrial fibrillation in the past decade, such as the convergent procedure and totally thoracoscopic ablation, and their outcomes relative to historic approaches such as the Cox maze procedure.

## INTRODUCTION

The development of atrial fibrillation (AF) surgery reflects a scientific process from bench to bedside, with rigorous animal model studies that were then slowly applied in human patients. We review this history, in particular discussing past procedures used to treat AF, moving then to discuss those used in contemporary practice. Next, we provide an over-view of surgical treatment for rarer arrhythmias, including inappropriate sinus tachycardia (IST) and Wolff–Parkinson–White (WPW) syndrome. This review focuses on surgical treatment for arrhyth-mias, rather than catheter ablation.

## ATRIAL FIBRILLATION: PAST SURGICAL TREATMENTS AND THEIR DEVELOPMENT

Atrial fibrillation surgery had its first major mile-stone in 1980 with the development of the left atrial isolation procedure. This procedure confined AF to the left atrium, while the rest of the heart remained in sinus rhythm. The procedure involves a left atri-otomy which is extended in one direction across Bachmann’s bundle to the mitral valve annulus, and in the other direction to the level of the coronary sinus and then endocardially to the mitral valve annulus at this location.^1^ This electrically separates the majority of the body of the left atrium from the remainder of the heart, given that valve tissue does not conduct AF. Unfortunately, since the left atrium remained in AF, there was an ongoing risk of systemic thromboembolism. However, as the rest of the heart continued in sinus rhythm, the cardiac output returned to normal provided that no signifi-cant diastolic dysfunction existed. This is because the right atrium and right ventricle had atrioven-tricular (AV) synchrony, resulting in a normal right-sided cardiac output. Under these conditions, left-sided cardiac output was also normal, despite the absence of left atrial contractility, because the left heart has been shown to adapt to the normal right-sided input and produce a normal left-sided output.^1^

In 1985, a second major advancement was made with the introduction by Guiraudon of the corridor procedure. The corridor procedure creates an elec-trical corridor reaching from the sinoatrial (SA) node to the AV node, allowing normal conduction of sinus rhythm from the atria to the ventricles. The corridor procedure achieves this by isolating a strip of atrium stretching between the SA node and the AV node.^1^ Despite this, the patient does not have AV synchrony restored as the majority of the left and right atria are not in continuity with this corridor, and therefore the hemodynamic abnormalities of AF persist. In addition, thromboembolic risk remains as AF continues in both atria. The main benefit of the corridor procedure is that a normal heart rate is restored without requirement for a permanent pacemaker as with AV node ablation.

The goal of AF surgery is therefore threefold: to restore AV synchrony and normal cardiac output; to eliminate the thromboembolic risk of AF; and to reduce symptoms arising from a rapid ventricular response with AF. To achieve this goal, sinus rhythm must be restored, and this goal spurred the develop-ment of surgical ablation for AF. In the 1980s, using canine models, a series of incisions were made in the atria to attempt to prevent the atria from fibrillating. Then in 1986 the first patient underwent the atrial transection procedure. This involved circumferential incisions in both atria connected by a transseptal incision.^1^ While this procedure resulted in sinus rhythm for 5 months, it ultimately failed. However, concurrent mapping studies both in a canine model and in humans with AF helped to establish that macro-reentry had a large role in AF. These studies ultimately led to the development of the still most effective therapy for AF, the Cox maze procedure.

The first maze procedure in a human patient was performed on September 25, 1987.^2^ Two iterations of maze-I quickly led to maze-III, which had the same effectiveness, though with reduced pacemaker risk, better sinus node function, and heart rhythm variability in response to exercise. The contempo-rary versions of the maze procedure are maze-III and maze-IV, with the latter using radiofrequency clamps and cryoablation to assist in complete abla-tion. There are several left atrial lesions: isolation of the left pulmonary veins, isolation of the right pul-monary veins (or a box lesion to encircle all four pulmonary veins), left superior vein to left atrial appendage lesion, closure or management of the left atrial appendage, epicardial coronary sinus lesion, and endocardial mitral isthmus line. There are three components to right atrial lesions: superior vena cava to inferior vena cava lesion, right atrial lesions to the tricuspid annulus in two spots, and a right atrial appendage lesion.^3^ While these are performed separately, it is important to remember that the left and right atria are one contiguous muscle electrical-ly, and therefore a complete maze procedure always involves lesions in both the left and right atria.

## ATRIAL FIBRILLATION: PRESENT AND FUTURE

The current status of AF involves several tiers of therapy, with a ladder of increasing efficacy. At the highest level of efficacy remains the maze procedure. This is performed with the use of a heart–lung machine (on pump), and can be done through a sternotomy, as well as minimally invasively with a small right thoracotomy. A large series of 133 patients who underwent stand-alone right mini-thoracotomy maze has shown a 79% rate of sinus rhythm off anti-arrhythmic medications (reported per Heart Rhythm Society guidelines) after this single operation, and a single stroke in 718 patient-years of follow-up.^4^ Moreover, 78% of patients in this series had long-standing persistent AF.

In the middle of the efficacy range is total thoracoscopic (TT) ablation, or, as it has come to be known, TT maze. This is a minimally invasive off-pump left atrial ablation. It involves bilateral pul-monary vein isolation with a bipolar radiofrequency clamp, and monopolar radiofrequency ablation of the roof and floor of the left atrium. Some surgeons will make the beginnings of a coronary sinus lesion, ablating from the floor lesion toward the coronary sinus, but this does not cross the coronary sinus or ablate it as this is not possible during this beating heart operation. The left pulmonary vein isolation is then connected to the base of the appendage, which is clipped. This clip has been shown to electrically isolate the appendage.^5^ The TT maze omits all the lesions of the right-sided maze procedure and does not provide a full left-sided lesion set, as the mitral isthmus line and coronary sinus line cannot be performed. Therefore, patients with a TT maze are traditionally sent for follow-up endocardial ablation. Results from a single busy center in 445 patients who completed the hybrid approach showed free-dom from AF without the use of anti-arrhythmic medications of 81%, 76%, and 58.5% at 1, 2, and 3 years;^5^ 7.5% required an electrophysiology study for atrial tachycardias. Therefore, by Heart Rhythm Society guidelines the success rate of 66% at 3 years that was cited in the paper was an overestimation.^6^

At the lower end of the efficacy range is the con-vergent operation, which involves a monopolar abla-tion of the back wall of the left atrium with a beating heart, followed by an electrophysiology study and ablation. The randomized CONVERGE trial was published in 2021 and randomized 153 patients with persistent or long-standing persistent AF 2:1 to hybrid convergent or to catheter ablation alone.^7^ Patients in the convergent arm underwent surgical ablation first, followed by endocardial ablation in standard fashion in order to complete isolation of the pulmonary veins—which is not possible during the surgical portion—and to treat gaps seen on electroanatomical mapping. In addition, a cavotri-cuspid isthmus ablation was performed. In the cath-eter ablation alone group, the pulmonary veins were isolated and a roofline performed, and a cavotricus-pid isthmus ablation was completed. Efficacy was defined as freedom from atrial tachyarrhythmia without new or increased dosage of a class I or III antiarrhythmic medication. The study revealed that the primary endpoint for success was reached by 67.7% of patients undergoing the convergent pro-cedure versus 50% of patients who had catheter ablation alone.^7^ Of note, these data do not meet the Heart Rhythm Society guideline definitions of abla-tion success, and therefore the true success of the convergent procedure was significantly lower at about 50%, since the primary efficacy endpoint could be achieved with the patient returning to an old dose of antiarrhythmic medication, which is not allowed to define success by Heart Rhythm Society guidelines. Initial meta-analysis had concluded that the convergent procedure was unsafe, with six trials including four from the United States showing a com-bined mortality rate of 1.7%, 75% of which were due to atrio-esophageal fistula.^8^ These series included patients treated from 2009 to 2014. The more recent CONVERGE trial data showed no mortality or atrio-esophageal fistula in 149 patients. The advantage of the convergent procedure compared to TT maze is that the former does not require single-lung ventila-tion, it is simpler to perform requiring less operator skill, and, in contemporary practice with esophageal temperature monitoring and increased awareness, seems to be associated with a reduction in esopha-geal injury. The primary drawback is that follow-up at 3 to 5 years is lacking, and it might be that TT maze is only marginally more effective than catheter ablation alone.

Future directions for AF surgery include the increased adoption of ablation during concomitant cardiac surgery, and development of new ablation devices for use in surgery. Another important future direction is increased adoption of AF ablation dur-ing cardiac surgery. The Society of Thoracic Sur-geons released expert consensus guidelines for sur-gical ablation for AF in 2017. These provided a class I, level A recommendation for surgical ablation at the time of concomitant mitral operations, and class I, level B recommendation for surgical ablation at the time of concomitant coronary bypass, aortic valve replacement, or combined coronary bypass and aortic valve replacement.^9^ Already at the time of publication of these guidelines, surgical ablation was on the rise with a 50% increase in ablation volumes over a study period ranging from 2011 to 2014.^10^ Subsequently, there has been increasing data to support that surgical ablation for AF might improve long-term survival. In a retrospective analysis from 2019 examining 20,407 consecutive coronary bypass or valve operations, 23.1% received ablation. A sub-stantial improvement in 5-year survival was seen in patients undergoing surgical ablation, with no dif-ference in perioperative outcomes.^11^ Meanwhile, more literature is emerging on the increased rate of ablations performed by cardiac surgeons. One series examined patients from 2014 to 2020 undergoing coronary bypass, aortic valve replacement, coronary bypass plus aortic valve replacement, isolated mitral operations, and mitral valve plus coronary opera-tions.^12^ Overall the rate of surgical ablation increased from 7.5% in the first quarter of 2014 to 40.9% in the first quarter of 2020. Patients undergoing mitral and mitral plus coronary operations now have a rate of surgical ablation of 66.7% and 50.0%, respectively. More encouragingly, younger surgeons are now more likely to perform ablation than older surgeons. There was an odds ratio of 0.71 for performance of surgical ablation for every 10-year increase in years since graduation from medical school. This demon-strates the increasing prevalence of surgical ablation of AF at the time of cardiac surgery.

With regard to new devices, recently a bipolar radiofrequency device called the EnCompass clamp (AtriCure, Mason, OH, USA), which can clamp both pulmonary veins as well as the back wall of the left atrium, has been released, allowing surgeons to cre-ate a box lesion set with one clamp application dur-ing concomitant open heart surgery.^13^ Long-term clinical outcomes have yet to be demonstrated, but devices such as this may further increase adoption of ablation by cardiac surgeons, although for now these can be applied only with the support of cardiopulmonary bypass.

Finally, in deciding on surgical treatment of patients with AF, it is helpful to have a framework for assessing patients. Surgeons will generally be confronted with patients with symptomatic AF who have failed antiarrhythmic medications and at least one catheter ablation. Hybrid procedures such as the convergent procedure and the TT maze can be considered for patients with a left atrial size less than 5.5 cm who have paroxysmal AF or a short duration of persistent AF.^14^ When patients have a left atrial size of more than 5.5 cm and a long duration of persistent AF, they are best managed with a complete Cox maze procedure,^14^ and the minimally invasive procedure offers a number of benefits, including published mortality rates below hybrid ablation and the highest efficacy rates seen in treatment of AF.^4^

## SURGICAL MANAGEMENT OF THE LEFT ATRIAL APPENDAGE

A final important direction will be a likely trend to increased management of the left atrial appendage at the time of cardiac surgery. In the LAOOS III trial published in 2021, 4,770 patients with AF and a CHA_2_DS_2_-VASc score of 2 or greater were random-ized to undergo or not undergo occlusion of the left atrial appendage at the time of cardiac surgery. Stroke or systemic embolism occurred in 4.8% of patients in the occlusion group and in 7.0% of pa-tients in the no occlusion group, and there were no major complications of left atrial appendage man-agement.^14^ Of note, the benefit of appendage occlu-sion was seen both in patients receiving chronic anticoagulation and in those who did not.^15^ Further, given the limitations of oral anticoagulation, appendage management at the time of surgery may provide a more durable solution to reduce the risk of stroke in AF. In particular, only 75% of patients on oral anticoagulation take more than 80% of prescribed doses, 25% interrupt it for invasive procedures, and 30% to 50% have stopped taking it at 3 years. With data from LAOOS III, it has become clear that all patients with AF and an elevated CHA_2_DS_2_-VASc score should have their atrial appendage occluded at the time of cardiac surgery.^15^

The trend to increased management of the left atrial appendage may soon apply to patients without AF undergoing cardiac surgery. In the ATLAS trial, 562 patients without preoperative AF were random-ized 2:1 to left atrial appendage exclusion or no left atrial appendage exclusion.^16^ In the exclusion group, there was a rate of 3.4% of thromboembolic events, versus 5.6% in the group without exclusion. This raises the prospect in the future of patients without AF having appendage management during cardiac surgery.^17^,^18^

## SURGICAL ABLATION FOR WOLFF–PARKINSON–WHITE SYNDROME

Wolff–Parkinson–White (WPW) syndrome was described in 1930, and Sealy and colleagues at Duke University (Durham, NC, USA) were the first to successfully divide an accessory pathway on May 2, 1968.^19^ Perhaps the largest series of patients with WPW undergoing surgical ablation was published in 1985 by Dr James Cox, inventor of the maze procedure.^20^ The main indication for surgery was medical refractoriness or drug intolerance (60%). The most common distribution of the accessory pathway(s) was 58% in the left free wall, and, of 149 accessory pathways, 148 were successfully divided. The classic approach to divide the accessory pathway is endocardial,^20^,^21^ but epicardial approaches supplemented by cryoablation have also been de-scribed.^21^ In fact, Sealy at Duke had used an epi-cardial approach for the first described case of surgically treated WPW. While there are too few patients for a trend to be discerned, some authors have suggested that the endocardial approach is more appropriate for left free wall pathways, while the epicardial approach may be more suitable for posterior septal pathways.^21^ Catheter ablation for accessory pathways in the AV junction was first introduced by Scheinman in 1981, and subsequent introduction of radiofrequency energy made cath-eter ablation standard of care for WPW.^19^

## SURGICAL ABLATION FOR INAPPROPRIATE SINUS TACHYCARDIA

Inappropriate sinus tachycardia is characterized by a fast resting heart rate and an abnormally exagger-ated rate response to exercise or stress. The stan-dard of care for this disorder is medical manage-ment. Beta-blockers are the main treatment but are often ineffective and can be poorly tolerated.^22^ Recently, the funny current inhibitor ivabradine has been studied in the treatment of IST and appears to be more effective than metoprolol.^23^ Some patients can require surgical ablation, and this can be done through sternotomy or a right thoracotomy. One of the largest series in the literature describes 18 pa-tients who underwent surgical ablation from 1987 to 2018.^24^ Ten patients had a sternotomy, while eight had a right thoracotomy. The key is to isolate the SA node with either surgical incision, cryoablation, or bipolar radiofrequency ablation. In this series, sinus tachycardia was eliminated in all patients, and long-term data were available for 17 of the 18 patients with mean follow-up of 11.4 years. At last follow-up, all patients remained 100% free from recurrent symptomatic IST. Therefore, while regarded as a therapy of last resort, surgical treatment of IST re-mains rewarding and effective in patients refractory to medical management.

## SURGICAL ABLATION FOR VENTRICULAR TACHYCARDIA

As with WPW syndrome, the surgical treatment of ventricular tachycardia (VT) has largely been replaced by catheter ablation. This is even more so for ischemic and scar-related VT. However, for some patients with scar-related VT with a non-ischemic etiology, the success of radiofrequency ablation is more variable.^25^ Many non-ischemic patients have a scar burden which is perivalvular and epicardial. These patients can even have localized epicardial scar seen by magnetic resonance imaging.^26^ A hybrid ablation can be performed with subxiphoid or left thoracotomy access to the pericardial space. Usually subxiphoid access is gained percutaneously as first described in 1996, but this can fail in up to 28% of patients, requiring a surgeon to gain surgical access to the pericardial space due to failure of percu-taneous access, or proximity of the VT focus to a coronary artery or phrenic nerve.^26^ Therefore the main role for surgeons in contemporary VT ablation is enabling epicardial ablation.

Finally, mitral valve prolapse is known to be associated with sudden cardiac death from VT, an entity termed arrhythmogenic mitral valve pro-lapse.^27^–^31^ Myocardial fibrosis is known to occur in one-third of patients with mitral valve prolapse. An imaging study from 2020 examined 20 patients with mitral valve prolapse and ventricular ectopy using simultaneous cardiac 18F FDG-PET and magnetic resonance imaging with late gadolinium enhance-ment: 85% of patients had evidence of PET positive lesions, and 70% of patients had PET positive lesions which also had late gadolinium enhancement. This study showed a relationship between degenerative mitral valve prolapse and myocardial injury and in-flammation.^28^ In particular, papillary muscle prema-ture ventricular contractions (PVCs) are common in patients with mitral valve prolapse, and these can trigger ventricular tachyarrhythmias. Ablation of the papillary muscles with cryoablation at the time of mitral valve repair has been reported, with localiza-tion of the papillary muscle origin of the PVC on the basis of a 12-lead electrocardiogram.^29^ In three patients with a high burden of PVCs, PVC burden was reduced by 97.9% with intraoperative cryoabla-tion. This might expand the role of surgical ablation in mitral valve repair to include papillary muscle ablation in properly selected patients.

## CONCLUSIONS

There is a rich history of ablation for arrhythmias in cardiac surgery. While surgical ablation for dis-orders such as WPW syndrome has been largely phased out, surgical ablation for AF remains on the rise and will continue to make an impact on this increasingly common arrhythmia.

## Abbreviations

AF: atrial fibrillation
AV: atrioventricular
IST: inappropriate sinus tachycardia
SA: sinoatrial
TT: total thoracoscopic
VT: ventricular tachycardia
WPW: Wolff–Parkinson–White.
